# Development of a prognostic risk score to predict early mortality in incident elderly Japanese hemodialysis patients

**DOI:** 10.1371/journal.pone.0302101

**Published:** 2024-04-11

**Authors:** Hirokazu Okada, Atsushi Ono, Koji Tomori, Tsutomu Inoue, Norio Hanafusa, Ken Sakai, Ichiei Narita, Toshiki Moriyama, Yoshitaka Isaka, Kei Fukami, Seiji Itano, Eiichiro Kanda, Naoki Kashihara

**Affiliations:** 1 Department of Nephrology, Saitama Medical University, Irumagun, Japan; 2 Department of Nephrology, SUBARU Health Insurance Association Ota Memorial Hospital, Ota, Japan; 3 Department of Medicine, Blood Purification, Tokyo Women’s Medical University, Tokyo, Japan; 4 Department of Nephrology, Toho University, Tokyo, Japan; 5 Division of Clinical Nephrology and Rheumatology, Niigata University Graduate School of Medical and Dental Sciences, Niigata, Japan; 6 Health and Counseling Center, Osaka University, Osaka, Japan; 7 Department of Nephrology, Osaka University Graduate School of Medicine, Osaka, Japan; 8 Department Medicine, Division of Nephrology, Kurume University School of Medicine, Fukuoka, Japan; 9 Department of Nephrology and Hypertension, Kawasaki Medical School, Kurashiki, Japan; 10 Department of Medical Science, Kawasaki Medical School, Kurashiki, Japan; 11 Geriatric Medical Center, Kawasaki Medical School, Okayama, Japan; The University of the West Indies, JAMAICA

## Abstract

**Background:**

Information of short-term prognosis after hemodialysis (HD) introduction is important for elderly patients with chronic kidney disease (CKD) and their families choosing a modality of renal replacement therapy. Therefore, we developed a risk score to predict early mortality in incident elderly Japanese hemodialysis patients.

**Materials and methods:**

We analyzed data of incident elderly HD patients from a nationwide cohort study of the Japanese Society for Dialysis Therapy Renal Data Registry (JRDR) to develop a prognostic risk score. Candidate risk factors for early death within 1 year was evaluated using multivariate logistic regression analysis. The risk score was developed by summing up points derived from parameter estimate values of independent risk factors. The association between risk score and early death was tested using Cox proportional hazards models. This risk score was validated twice by using an internal validation cohort derived from the JRDR and an external validation cohort collected for this study.

**Results:**

Using the development cohort (n = 2,000), nine risk factors were retained in the risk score: older age (>85), yes = 2, no = 0; sex, male = 2, female = 0; lower body mass index (<20), yes = 2, no = 0; cancer, yes = 1, no = 0; dementia, yes = 3, no = 0; lower creatinine (<6.5 mg/dL), yes = 1, no = 0; lower albumin (<3.0 g/dL), yes = 3, no = 0; normal or high calcium (≥8.5 mg/dL), yes = 1, no = 0; and higher C reactive protein (>2.0 mg/dL), yes = 2, no = 0. In the internal and external validation cohorts (n = 739, 140, respectively), the medium- and high-risk groups (total score, 6 to 10 and 11 or more, respectively) showed significantly higher risk of early death than the low-risk group (total score, 0 to 5) (p<0.001).

**Conclusion:**

We developed a prognostic risk score predicting early death within 1 year in incident elderly Japanese HD patients, which may help detect elderly patients with a high-risk of early death after HD introduction.

## Introduction

Hemodialysis (HD) is an essential life-saving treatment for patients with end-stage kidney disease (ESKD); however, its usefulness is not being fully utilized in an increasing number of elderly patients with systemic complications such as ischemic heart disease, cognitive impairment, and frailty, who often experience rapid decline in activity of daily living (ADL) and deterioration in quality of life (QOL) after HD introduction [1,2]. Patients and their families sometimes regret the decision to introduce HD or wish to discontinue it. Although the long-term prognosis of maintenance HD patients in Japan is among the best in the world, the short-term prognosis in the first 4 months after introduction of HD is comparable to that in other countries [3]. Especially for patients aged 80 years or older, the mortality rate in the first 12 months after introduction reaches 30%, and about half of these patients die within 3 months after introduction [4]. When choosing a modality in renal replacement therapy (RRT), it is desirable that patients, their families, and medical professionals, including physicians, meet for shared decision-making (SDM), where all treatment options of RRT, including forgoing dialysis and conservative kidney management (CKM), are presented and the outcome of each option should be concretely explained [5,6]. For elderly patients and their families, information about short-term rather than long-term prognosis is more informative when choosing between maintenance dialysis and CKM. In foreign countries where CKM has been presented at SDM sessions for a longer time than in Japan, many prognostic models predict life expectancy in elderly patients with CKD who opt for HD [7–22]. Unfortunately, there have been very few reports regarding that for CKM [23].

In this study, we attempted to identify risk factors associated with early mortality within 1 year of HD introduction in elderly patients with CKD aged 75 years or older, and to develop a prognostic equation for life expectancy using the results of a nationwide cohort study of incident HD patients in Japan in 2006 and 2007.

## Materials and methods

### Development and internal validation cohort

Japanese Society of Dialysis Therapy (JSDT) has been conducting annual surveys of >99% of all HD facilities in Japan: JSDT Renal Data Registry (JRDR). In 2006 and 2007, JRDR collected incident HD patient data just prior to the time of HD introduction, and stored follow-up data on post-introduction survival for all the patients. Therefore, we used JRDR data from 2006 and 2007 to create a prognostic risk score to predict early mortality in incident elderly HD patients.

**Fig 1:**
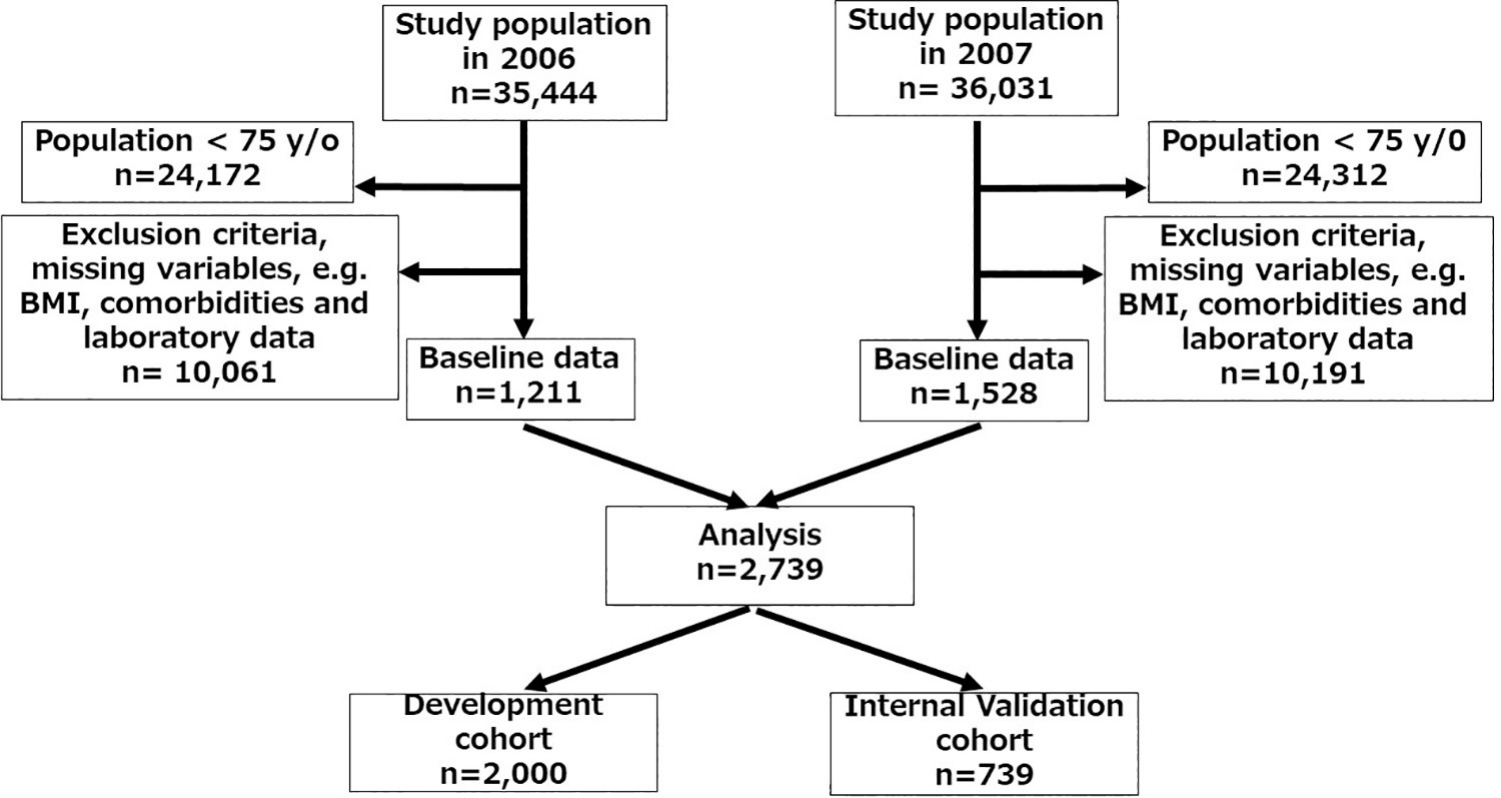
Flow diagram of the study population.

We obtained the anonymous data from JRDR in October, 2019. All 2,739 patients who initiated incident HD in 2006 and 2007 were enrolled using the following exclusion criteria (Fig 1): age <75 years, withdrawal from HD because of kidney transplantation and transition to peritoneal dialysis (PD), and missing values such as laboratory data. The included patients were randomly classified into two groups to obtain datasets of the development cohort (2,000) and internal validation cohort (739).

### External validation cohort

Between March 2020 and March 2021, the incident elderly HD patients aged 75 or older (n = 140) were enrolled in a prospective, external validation cohort at six university hospitals scattered geographically in Japan (Saitama Medical University, Niigata University, Toho University, Osaka University, Kawasaki Medical School, Kurume University), and followed-up until March 2022.

### Patient data

The datasets of the development and internal validation cohorts and the external cohort contained the following variables: sex; age; body mass index (BMI); comorbidities of cardiovascular disease (CVD) consisting of congestive heart failure and coronary arterial disease, any cancer and dementia determined by the attending physician to have any cognitive impairment; diabetic kidney disease (DKD) as a primary kidney disease; serum albumin, urea nitrogen (UN), creatinine (Cr), inorganic phosphorus (Pi), calcium (Ca), and C reactive protein (CRP) levels; hemoglobin (Hb) level; and presence or absence of arterio-venous fistula (AVF).

For the convenience of clinical practical use, all continuous variables were converted to binary categorical variables for prognostic risk score construction; age (super-elderly, defined as ≥ 85 years (yes = 1, no = 0)), BMI (underweight, defined as < 20 (yes = 1, no = 0)), UN (low, defined as < 65 mg/dL (yes = 1, no = 0)), Cr (low, defined as < 6.5 mg/dL (yes = 1, no = 0)), Albumin (low, defined as < 3.0 g/dL (yes = 1, no = 0)), Ca (normal or high, defined as ≥ 8.5 mg/dL (yes = 1, no = 0)), Pi (high, defined as ≥ 3.5 mg/dL (yes = 1, no = 0)), CRP (high, defined as ≥ 2.0 mg/dL (yes = 1, no = 0)) and Hb (low, defined as < 10 g/dL (yes = 1, no = 0)). The cutoff values used to convert UN and Cr to binary categories were based on the average estimated glomerular filtration rate (eGFR) (5 mL/min/1.73m^2^) at the time of incident HD in Japan [24]. Others were determined with reference to the common/target values for patients undergoing maintenance HD [25,26].

The primary outcome was early death, defined as all-cause death with 1 year of HD introduction. The JRDR data contained dates of death for all patients who died. For the external validation cohort, the dates of death were recorded. If no outcomes were observed within the follow-up period, the observation data were treated as censored data.

### Statistical analysis

Normally distributed, continuous variables are presented as mean ± standard deviation (SD); otherwise, the median and interquartile range are presented. Intergroup comparisons of variables were performed using Chi-squared test, t-test, and Mann-Whitney U test, as appropriate.

The analyses were conducted using EZR version 1.63 (Saitama Medical Center, Jichi Medical University, Saitama, Japan), which is a graphical user interface for R (The R Foundation for Statistical Computing, Vienna, Austria, version 2.13.0) [27]. Statistical significance was defined as p<0.05.

### Development of risk score

Using the development cohort dataset, univariate and multivariate logistic regression analyses were performed to identify risk factors significantly and independently associated with early death within 1 year in incident HD patients. Collinearity was tested using variance inflation factor (VIF), and variables with VIF≥2 were omitted because of collinearity.

A weighted score proportional to the smallest parameter estimate of the independent risk factors was assigned to each categorical index, which was rounded to the nearest integer. For each patient, the risk score was calculated as the sum of the points.

Based on categorical criteria for the risk score, the patients were divided into three risk groups using Kaplan-Meier survival curves: low-, medium-, and high-risk. Then, the survival probabilities of the groups were compared using log-rank test. Moreover, the risk of the outcome was compared between risk groups by Cox proportional hazards models adjusted for baseline characteristics not included in the risk score. The proportional hazards assumption was graphically verified using log-log plots. Results of statistical analyses are presented as hazard ratios (HRs) with 95% confidence of intervals (CI), as appropriate.

### Validation of risk score

The accuracy of the prediction of the outcome of the risk score was evaluated on the basis of area under the receiver operating characteristic (ROC) curve (AUC) and c-statistics using datasets from the internal validation as well as the external validation cohort. Additionally, the risk score was validated using Kaplan-Meier survival curves and Cox proportional hazard models as described above.

### Ethics

The development and internal validation cohort study was approved by the ethics committee of JSDT (JSDT No.2) and was exempt from the need to obtain informed consent from participants. The external validation cohort study was approved by the Institutional Review Board of Saitama Medical University (19091.01) as a base facility for this multi-center collaborative research, and we obtained informed consent from all the participants.

All data were analyzed anonymously. The study was performed in accordance with the Declaration of Helsinki (revised 2013) and the ethical guidelines for medical and health research involving human subjects by the Japanese Ministry of Health, Labour and Welfare (revised 2023).

## Results

### Baseline characteristics

The final study population consisted of 2,739 patients (Fig 1), and 424 deaths occurred within 1 year of the follow-up period. Causes of death included infection in 102 patients, congestive heart failure in 80, malignancy in 35, stroke in 30, cardiovascular disease in 14, and unknown in 163. Baseline demographic and clinical characteristics of the study population by primary endpoint are shown in Table 1.

**Table 1:** Comparison of patient characteristics and laboratory data of the study population at incident hemodialysis by primary endpoint.

Basic characteristics	All	Survive ≥ 1 year	Death <1 year	p-value
**N**	**2,739**	**2,315**	**424**	
**Sex (Male) (%)**	**1,570 (57.3)**	**1.317 (56.8)**	**253 (59.7)**	**0.31**
**Age (years)**	**80 (77, 83)**	**80 (77, 83)**	**81 (78, 85)**	**<0.001**
**BMI (kg/m2)**	**21.8±3.9**	**22.0±3.9**	**20.9±3.8**	**<0.001**
**Primary kidney disease**				
**DKD (%)**	**1,092 (39.9)**	**936 (40.4)**	**156 (36.8)**	**0.16**
**CGN (%)**	**633 (23.1)**	**531 (22.9)**	**102 (24.1)**	**0.62**
**PKD (%)**	**43 (1.6)**	**12 (0.5)**	**6 (1.4)**	**1.00**
**Nephrotic (%)**	**8 (0.3)**	**8 (0.3)**	**0**	**0.62**
**Others (%)**	**963 (35.1)**	**836 (35.9)**	**160 (37.7)**	**0.25**
**Laboratory data**				
**UN (mg/dl)**	**86.4±30.4**	**86.4±30.0**	**86.9±32.4**	**0.77**
**Cr (mg/dl)**	**7.3±2.9**	**7.4±2.8**	**6.8±3.2**	**<0.001**
**Albumin (g/dL)**	**3.2±0.6**	**3.3±0.6**	**3.0±0.6**	**<0.001**
**Ca (mg/dl)**	**7.9±1.0**	**8.0±1.0**	**8.0±1.2**	**0.22**
**Pi (mg/dl)**	**5.3±1.6**	**5.4±1.6**	**4.9±2.0**	**0.06**
**CRP (mg/dl)**	**0.46 (0.1, 2.1)**	**0.34 (0.1, 1.6)**	**1.72 (0.4, 4.8))**	**<0.001**
**Hb (g/dl)**	**8.4±1.5**	**8.4±1.5**	**8.4±1.6**	**0.99**
**AVF (%)**	**1,465 (53.5)**	**1,332 (57.5)**	**133 (31.3)**	**<0.001**
**Comorbidities**				
**CVD (%)**	**255 (9.3)**	**206 (8.9)**	**49 (11.6)**	**0.08**
**Cancer (%)**	**196 (7.2)**	**149 (6.4)**	**47 (11.1)**	**0.001**
**Dementia (%)**	**453 (16.5)**	**323 (14.0)**	**130 (30.7)**	**<0.001**
**HD Duration (month)**			**6.1±3.7**	

The study population was randomly assigned into two cohort groups: one group for the development cohort and the other for the internal validation cohort (Fig 1). No significant differences in baseline patient characteristics between the datasets of the development and internal validation cohorts were observed (Table 2). The baseline patient characteristics of the external validation cohort are shown in Table 3.

**Table 2:** Comparison of patient characteristics and laboratory data of the study population randomly divided to the development and internal validation cohorts.

Baseline characteristics	All	Development	Internal Validation	p-value
**N**	**2,739**	**2000**	**739**	
**Sex (Male) (%)**	**1,570 (57.3)**	**1153 (57.7)**	**417(56.4)**	**0.57**
**Age (years)**	**80 (77, 83)**	**80 (77, 83)**	**80 (77, 83)**	**0.32**
**BMI (kg/m2)**	**21.8±3.9**	**21.9±4.0**	**21.8±3.6**	**0.90**
**Primary kidney disease**				
**DKD (%)**	**1,092(39.9)**	**811(40.6)**	**281(38.9)**	**0.24**
**Laboratory data**				
**UN (mg/dl)**	**86.4±30.4**	**86.8±30.2**	**85.6±31.0**	**0.37**
**Cr (mg/dl)**	**7.3±2.9**	**7.3±2.9**	**7.3±2.8**	**0.49**
**Albumin (g/dL)**	**3.2±0.6**	**3.2±0.6**	**3.2±0.6**	**0.76**
**Ca (mg/dl)**	**7.9±1.0**	**8.0±1.1**	**8.0±1.1**	**0.18**
**Pi (mg/dl)**	**5.3±1.6**	**5.3±1.6**	**5.3±1.6**	**0.55**
**CRP (mg/dl)**	**0.46 (0.1, 2.1)**	**0.46 (0.1, 2.08)**	**0.46 (0.1, 2.38)**	**0.36**
**Hb (g/dl)**	**8.4±1.5**	**8.3±1.5**	**8.4±1.4**	**0.18**
**Absence of AVF(%)**	**1,274(46.5)**	**928(46.4)**	**346(46.8)**	**0.86**
**Comorbidities**				
**CVD (%)**	**255 (9.3)**	**192 (9.6)**	**63 (8.5)**	**0.42**
**Cancer (%)**	**196 (7.2)**	**146 (7.3)**	**50 (6.8)**	**0.68**
**Dementia (%)**	**453 (16.5)**	**342 (17.1)**	**111 (15.0)**	**0.20**
**Death**<**1 year(%)**	**424 (15.5)**	**313(15.7)**	**111(15.0)**	**0.72**

**Table 3:** Patient characteristics and laboratory data of the external validation cohort.

Baseline characteristics.	
**N**	**140**
**Gender (Male) (%)**	**87 (62.1)**
**Age (years)**	**81 (78, 84)**
**BMI (kg/m2)**	**22.4±3.9**
**Primary kidney disease**	
**DKD (%)**	**55 (39.3)**
**Laboratory data**	
**UN (mg/dl)**	**86.1±27.1**
**Cr (mg/dl)**	**7.3±2.2**
**Albumin (g/dL)**	**3.2±0.6**
**Ca (mg/dl)**	**8.5±0.8**
**Pi (mg/dl)**	**5.4±1.4**
**CRP (mg/dl)**	**0.32 (0.1, 1.09)**
**Hb (g/dl)**	**10.1±6.9**
**Absence of AVF (%)**	**47 (33.6)**
**Comorbidities**	
**CVD (%)**	**34 (24.3)**
**Cancer (%)**	**15 (10.7)**
**Dementia (%)**	**55 (39.3)**
**Death**<**1 year (%)**	**18 (12.9)**

### Independent risk factors for early death within 1 year

Using the dataset of the development cohort, univariate logistic regression analyses were performed, and some of variables were significantly associated with early death within 1 year in incident elderly HD patients (Table 4). To create the risk score, subsequent multivariate logistic regression analyses were performed, and variables such as older age (>85), male sex, lower BMI (<20), cancer (Yes), dementia (Yes), lower Cr (<6.5), lower albumin (<3.0), normal or high Ca (≥8.5), and higher CRP (>2.0) were found to be significantly and independently associated with early death (Table 5). No collinearity was seen for all the variables being used.

**Table 4:** Results of univariate logistic regression analysis of the development cohort.

Baseline characteristics	OR	95%CI	p-value
**Age (years) ≧85**	**2.25**	**1.72, 2.95**	**<0.001**
**Sex (Male)**	**1.36**	**1.06, 1.75**	**0.015**
**BMI (kg/m2) <20**	**1.96**	**1.53, 2.51**	**<0.001**
**DKD: Yes**	**0.94**	**0.73, 1.20**	**0.623**
**Laboratory variables**			
**UN (mg/dl) <65**	**1.13**	**0.85, 1.49**	**0.406**
**Cr (mg/dl) <6.5**	**1.57**	**1.23, 2.00**	**<0.001**
**Albumin (g/dL) <3.0**	**2.67**	**2.09, 3.42**	**<0.001**
**Ca (mg/dl) ≥ 8.5**	**1.08**	**0.83, 1.42**	**0.563**
**Pi (mg/dl) ≥ 3.5**	**0.55**	**0.39, 0.80**	**0.001**
**CRP (mg/dl) ≧2.0**	**2.96**	**2.30, 3.80**	**<0.001**
**Hb (g/dl) <10**	**0.92**	**0.65, 1.29**	**0.617**
**Absence of AVF no**	**3.39**	**2.61, 4.41**	**<0.001**
**Comorbidities**			
**CVD yes**	**1.28**	**0.87, 1.87**	**0.215**
**Cancer yes**	**1.79**	**1.20, 2.67**	**0.005**
**Dementia yes**	**2.80**	**2.13, 3.69**	**<0.001**

**Table 5:** Results of multivariate logistic regression analysis of the development cohort.

Variables	Parameter estimates	aOR (95%CI)	p-value
**Age (years) ≥ 85**	**0.733**	**2.08 (1.54, 2.81)**	**<0.001**
**Sex (Male)**	**0.520**	**1.68 (1.27, 2.22)**	**<0.001**
**BMI (kg/m2) < 20**	**0.632**	**1.88 (1.43, 2.47)**	**<0.001**
**Primary disease: DKD yes**	**-0.095**	**0.91 (0.69, 1.19)**	**0.489**
**Laboratory data**			
**UN (mg/dl) < 65**	**-0.036**	**0.97 (0.70, 1.34)**	**0.828**
**Cr (mg/dl) < 6.5**	**0.302**	**1.35 (1.02, 1.80)**	**0.037**
**Albumin (g/dL) < 3.0**	**0.771**	**2.16 (1.62, 2.85)**	**<0.001**
**Ca (mg/dl) ≥ 8.5**	**0.331**	**1.39 (1.02, 1.90)**	**0.035**
**Pi (mg/dl) ≥ 3.5**	**-0.11**	**0.90 (0.59, 1.36)**	**0.605**
**CRP (mg/dl) ≥ 2.0**	**0.743**	**2.10 (1.59, 2.78)**	**<0.001**
**Hb (g/dl) < 10**	**-0.060**	**0.94 (0.64, 1.38)**	**0.760**
**Absence of AVF no**	**0.963**	**2.62 (1.97, 3.48)**	**<0.001**
**Comorbidities**			
**CVD yes**	**0.107**	**1.11 (0.73, 1.69)**	**0.619**
**Cancer yes**	**0.451**	**1.57 (1.01, 2.44)**	**0.045**
**Dementia yes**	**0.798**	**2.22 (1.65, 3.00)**	**<0.001**

Abbreviation: aOR, adjusted odds ratio.

### Development of risk score

The score point was determined for each risk factor (Table 6).

**Table 6:** Parameter estimates of the independent risk factors and risk score points.

Variables	Parameter estimates	Ratio	Risk score point
**Age (years) ≥ 85**	**0.733**	**2.42**	**2**
**Sex (Male)**	**0.520**	**1.72**	**2**
**BMI (kg/m2) < 20**	**0.632**	**2.10**	**2**
**Cr (mg/dl) < 6.5**	**0.302**	**1.0**	**1**
**Albumin (g/dL) < 3.0**	**0.771**	**2.55**	**3**
**Ca (mg/dl) ≥ 8.5**	**0.331**	**1.10**	**1**
**CRP (mg/dl) ≥ 2.0**	**0.743**	**2.46**	**2**
**Absence of AVF no**	**0.963**	**3.19**	**3**
**Cancer yes**	**0.451**	**1.49**	**1**
**Dementia yes**	**0.798**	**2.64**	**3**

Risk score = older age + male sex + low BMI + low Cr + low albumin + normal or high Ca + high CRP + absence of AVF-no + cancer-yes + dementia-yes.

Older Age, yes = 2, no = 0; sex, male = 2, female = 0; low BMI, yes = 2, no = 0; low Cr, yes = 1, no = 0; low albumin, yes = 3, no = 0; normal or high Ca, yes = 1, no = 0; high CRP, yes = 2, no = 0; absence of AVF; no = 3, yes = 0; cancer, yes = 1, no = 0; dementia, yes = 3, no = 0.

**Fig 2:**
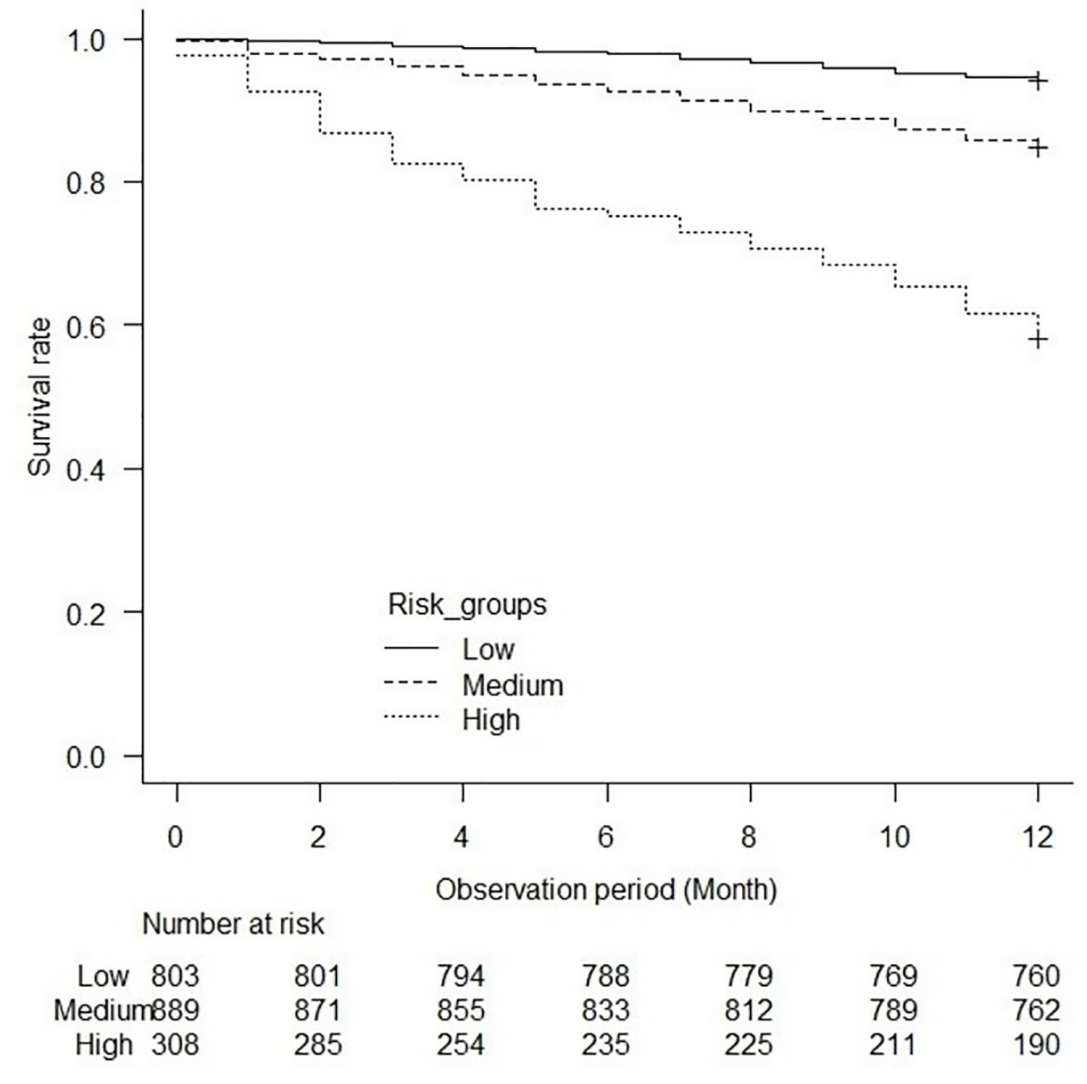
Association between the risk groups and mortality in the development cohort. The Kaplan-Meier survival curves show a significant difference in mortality between the risk groups (log-rank test, p<0.001).

The patients were categorized into three groups based on the risk score: low-risk, 0 to 5; medium-risk, 6 to 10; high-risk, 11 or higher. The Kaplan-Meier survival curve showed a significant difference between the groups (log-rank test, p<0.001) (Fig 2). The medium- and high-risk groups showed higher risks of early death within 1 year than the low-risk group (Table 7).

**Table 7:** Risk groups and risk of early death within 1 year in the development cohort.

Risk groups	HR	aHR
Low-risk group	Reference	Reference
**Medium-risk group**	**2.69** **(95CI, 1.94, 3.74; p<0.001)**	**2.67** **(95%CI, 1.92, 3.71; p<0.001)**
**High-risk group**	**8.79** **(95CI, 6.31, 12.24; p<0.001)**	**8.63** **(95CI, 6.18, 12.07; p<0.001)**

Abbreviation: aHR, adjusted hazard ratio.

### Validation of risk score

**Fig 3:**
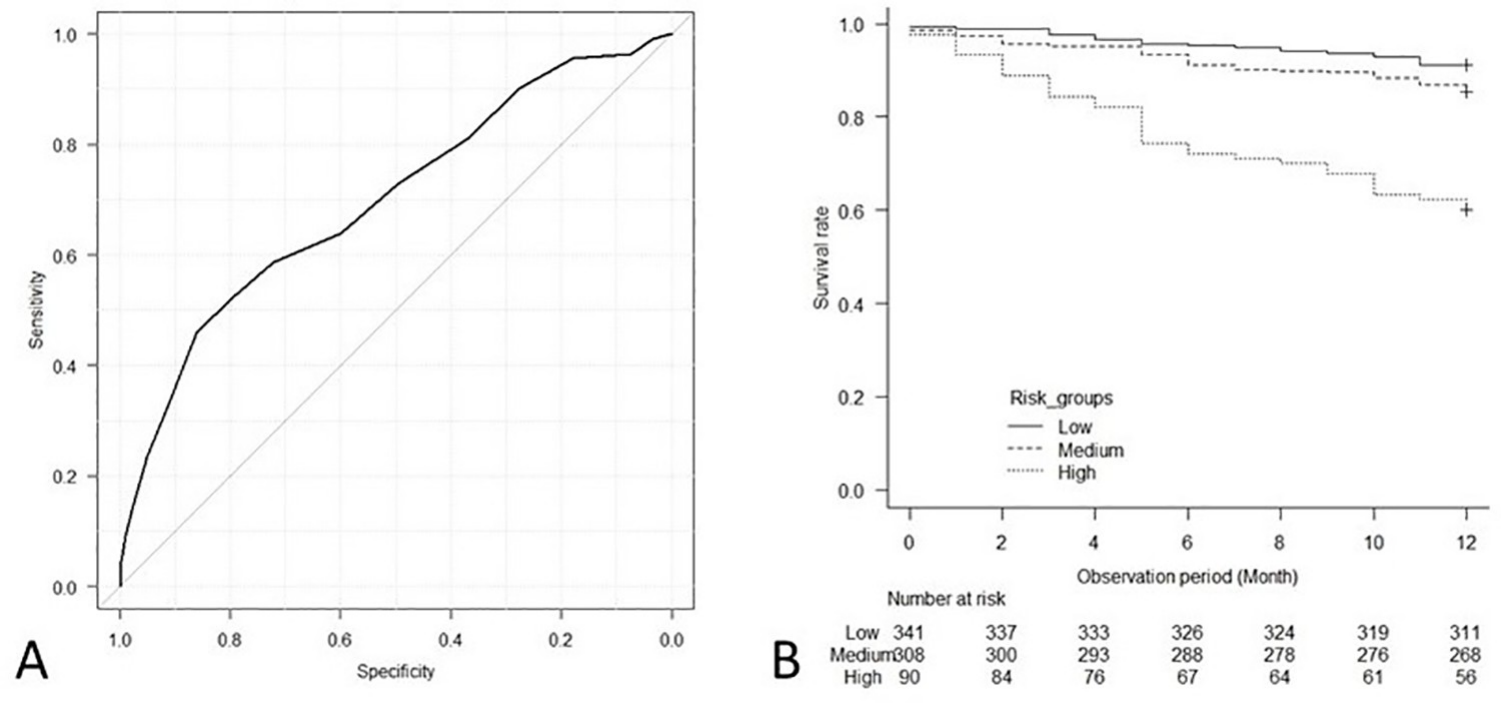
The ROC curve of the prognostic risk score for the prediction of early death within 1 year (A), and association between risk groups and mortality in the internal validation cohort (B). (A) The prognostic risk score showed high accuracy for the prediction of the outcome in the internal validation cohort; c-statistics, 0.70 (95% CI, 0.64, 0.75). (B) The Kaplan-Meier survival curves show a significant difference in mortality between the risk groups (log-rank test, p<0.001).

**Fig 4:**
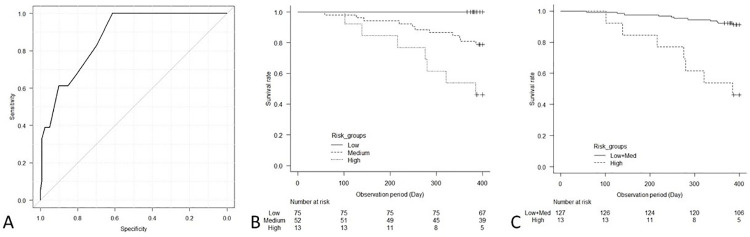
The ROC curve of the prognostic risk score for the prediction of early death within 1 year (A), and association between risk groups and mortality in the external validation cohort: Among three risk groups (B), among two risk groups (C). (A) The prognostic risk score showed high accuracy for the prediction of the outcome in the external validation cohort; c-statistics, 0.87 (95% CI, 0.80, 0.94). (B and C) In both analysis, Kaplan-Meier survival curves show significant differences in mortality between the risk groups (log-rank test, p<0.001).

This prognostic risk score showed high accuracy for the prediction of the outcome in the internal validation cohort; c-statistics, 0.70 (95% CI, 0.64, 0.75), as well as in the external validation cohort; c-statistics, 0.87 (95% CI, 0.80, 0.94) (Figs 3A and 4A). Additionally, the Kaplan-Meier survival curves for early death within 1 year showed significant differences between the groups in the internal validation cohort (log-rank test, p<0.001) and in the external validation cohort (log-rank test, p<0.001) (Figs 3B and 4B). In the internal validation cohort, the risks of early death in the medium- and high-risk groups were significantly higher than those in the low-risk group according to Cox proportional hazards models (Table 8).

**Table 8:** Risk groups and risk of early death within 1 year in the internal validation cohort.

Risk groups	HR	aHR
Low-risk group	Reference	Reference
**Medium-risk group**	**1.71** **(95CI, 1.08, 2.71; p = 0.02)**	**1.63** **(95%CI, 1.03, 2.60; p = 0.04)**
**High-risk group**	**5.53** **(95CI, 3.41, 8.99; p<0.001)**	**5.21** **(95CI, 3.18, 8.53; p<0.001)**

Since no events were observed in the low-risk group of the external validation cohort, the low- and the medium-risk groups were combined and used as the reference (Fig 4C and Table 9). The high-risk group showed approximately eight-times higher risk compared to the low-medium risk group.

**Table 9:** Risk groups and risk of early death within 1 year in the external validation cohort.

Risk groups	HR	aHR
Low and Medium-risk group	Reference	Reference
**High-risk group**	**8.31** **(95CI, 3.21, 21.52; p<0.001)**	**8.02** **(95CI, 2.65, 24.28; p<0.001)**

## Discussion

In this study, we used data from cross-sectional and subsequent longitudinal surveys of incident HD patients in Japan in 2006 and 2007. We aimed to develop prognostic risk scores for predicting early mortality within 1 year after HD introduction in elderly patients with CKD aged 75 years or older. Robust results were observed in internal validation using an internal validation cohort and external validation using a prospective cohort of incident HD elderly patients from 2020 to 2021. These results suggest that this prognostic risk score can at present be used reliably to predict early mortality after HD introduction in elderly Japanese ESKD patients, when patients, their families, and health professionals are making SDM sessions for a choice of modality in RRT. However, as mentioned in the Introduction, there are very few scores to estimate the life expectancy of elderly ESKD patients who choose PD or CKM. Therefore, it is important to note that presenting only the life expectancy in case of HD without mentioning those in cases of PD and CKM may be a biased guidance.

In the 1990s, it became common for patients to be offered the option of not receiving dialysis or CKM during SDM sessions [5,6,28]. Since it is necessary to provide information on patient prognosis after the introduction of RRT [5,6], various attempts to predict prognosis have been reported. Even if we limit articles published since 2000 that deal with prognostic models that are still expected to be useful in terms of life expectancy, we found 21 articles that analyzed risk factors just prior to HD introduction as explanatory variables and early death within 1 year after HD introduction as an objective variable [4,7–22,29–32].

The groups of explanatory variables that were independently and significantly related to the early death were demographic data (age, sex, BMI), laboratory data (serum albumin, serum creatinine, eGFR, blood hemoglobin, CRP), therapeutic drugs, underlying diseases causing CKD, and comorbidities (CVD, malignancy, obstructive lung disease). Older age, male sex, high serum CRP levels, and comorbidity with cancer have been reported as risk factors for early death [4,8–22,29,31,32], and are consistent with the clinical experience, which seems satisfactory. Additionally, variables such as BMI and serum albumin, which are indicators of nutritional status, have been discussed in many reports [4,7,9–11,13,14,16,17,19,20,22,30–32]. HD introduction in the absence of AVF is also considered to lead to a significant risk of early death in many reports, and HD introduction in the absence of AVF may be used as an index that includes multiple risks of mortality [4,8–10,13,14,20,22,31]. All these are significant risk factors in our prognostic risk score. On the other hand, it is not clear why normal or high serum Ca levels remained a risk factor [32], and the possibility that they might be associated with undiagnosed malignancy cannot be ruled out. In addition, the reason why either DKD or CVD, which was often reported as a risk factor [5–18,20,22,29,32], was not one in this study may be that patients with DKD and/or CVD with poor general condition were not introduced to HD and not included in the study population.

In recent years, an increasing number of reports have demonstrated prognostic factors related to aging, such as physical impairment, cognitive impairment, poor nutrition, frailty, and fall episodes, as explanatory variables in predicting life expectancy of dialysis patients [33–35]. These risk factors are likely confounding because they are closely related to each other, and except for poor nutrition, they are rarely employed as independent explanatory variables in the same prognostic model. Serum creatinine as laboratory data is a significant explanatory variable in several prognostic models, but each report shows conflicting associations between residual renal function at dialysis introduction and early death within 1 year after introduction [4,8,14–17,20,29,30,32,36]. Among them, one report showed that when low serum creatinine, which indicates early introduction of dialysis, was a significant risk factor for death, such significance disappeared when adjusted for frailty, suggesting that frailty is strongly associated with death [36]. Frailty is strongly related to the prognosis of dialysis patients as a composite index including multiple risks of mortality [37], and it is desirable to develop improved frailty assessment criteria for patients with CKD [38]. Low serum creatinine is also a risk factor in our prognostic risk score and may be associated with sarcopenia, a precursor condition of frailty. Most of the prognostic models for aging-related factors have been reported from foreign countries, but there have been several reports from Japan on the degree of dependence on nursing care related to physical dysfunction [4,29,30,32]. Although cognitive impairment has not been addressed extensively [17], we employed dementia as a candidate risk factor to be significantly associated with early death in our prognostic risk score.

A systematic review on the prediction of short- to long-term life expectancy in incident HD patients was reported by Anderson et al. in 2019 [39]. Among their results, the ROC curves with the largest AUC as discriminative of life expectancy were the Ivory index and the Obi index [16,17], both of which are useful with respect to predicting early death within 1 year. The latter in particular has shown robustness in external validation cohorts [17]. (http://www.dialysisscore.com/) The AUC of the ROC curve of our prognostic risk score were as discriminative as those of these indices.

The prognostic model of Inaguma et al. for Japanese patients is superior because of the size of the study population used to construct the prognostic model, the diversity of explanatory variables employed, and the high discriminative power for predicting early death within 1 year of HD introduction [32]. The difference between our risk score and theirs is that their model was based on the data collected from a limited region of Japan, while ours is based on national data. Additionally, the reliability of our prognostic risk score is further enhanced by the fact that a recent prospective cohort was constructed using data from all of Japan, and external validation was conducted using this cohort.

There are several limitations to this study. First, there may be selection bias due to the removal of a large number of patients with missing data from consideration. Second, the sample size became smaller because of the restriction of age and RRT modality. However, the number of HD patients who progressed to kidney transplant or PD was small and was removed from the study to make it easier to interpret the results. Third, although data were collected on important complications such as fluid overload, chronic pulmonary or liver disease, they could not be analyzed as candidate risk factors due to missing data or unknown responses. Fourth, although electrolytes other than Ca and Pi, medications, physical findings (e.g., blood pressure and physical function) and social settings are good candidate risk factors, those were not collected and thus not examined as risk factors. Therefore, this score could not be compared with existing models such as the Ivory index, Obi index and Inaguma et al. score [16,17,32]. The fifth is that this study did not examine ESKD patients who were not introduced to HD. Elderly ESKD patients who were introduced to HD are likely to be healthier than those not introduced. It is possible that DKD and CVD were not included as variables in the prognostic risk score because high-risk patients with DKD or CVD were not introduced to HD. Therefore, our prognostic risk score should be used with caution when generalizing to the entire population of elderly ESKD patients. Sixth, some information was lost because continuous variables were converted to binary categorical variables to create a clinically usable score. Seventh, there could be bias due to unintended confounding for an observational study. Eighth, because the observed endpoint was all-cause mortality, it was not possible to evaluate ADL prognosis and QOL, such as frail and bedridden patients.

In conclusion, we used nationwide cohort data to identify the major risk factors for early death within 1 year in incident elderly Japanese HD patients and developed a prognostic risk score in this study. The robustness of this prognostic risk score was confirmed by data from an internal validation cohort and a more recent prospective cohort, and future clinical applications are anticipated. In addition to the information provided by this prognostic risk score, we hope to provide information on the prognostic value of choosing PD or CKM, which will facilitate the decision making for elderly ESKD patients and their families regarding RRT modalities at SDM sessions.
